# Effects of legume-diet and sex of ducks on the growth performance, physicochemical traits of meat and fatty acid composition in fat

**DOI:** 10.1038/s41598-020-70508-x

**Published:** 2020-08-10

**Authors:** Emilia Kowalska, Joanna Kucharska-Gaca, Joanna Kuźniacka, Jakub Biesek, Mirosław Banaszak, Marek Adamski

**Affiliations:** Department of Animal Breeding, Faculty of Animal Breeding and Biology, UTP - University of Science and Technology in Bydgoszcz, Mazowiecka 28, 85-084 Bydgoszcz, Poland

**Keywords:** Zoology, Fatty acids

## Abstract

Duck meat enjoys growing popularity among consumers. Alternative protein sources to soybean are being investigated to eliminate genetically modified components from the poultry’ diet. The aim of this study was to compare growth performance, quality of meat, and fatty acid composition in subcutaneous and abdominal fat from ducks fed a diet based on yellow lupin and rapeseed meal, sources of protein alternative to soybean meal (SBM). Ducks were allocated to different dietary treatment groups and reared for 8 weeks (N = 102 per group). Group A received a diet based on SBM, while group B was fed a diet based on yellow lupin with the addition of rapeseed meal. Both groups were divided into two subgroups, of male and female birds. Growth performance parameters and zoometric traits of ducks were monitored during the growth period. After 8 weeks selected birds were slaughtered and dissected (N = 10 per group). Carcass composition was calculated and selected traits of meat quality important for further processing were analysed. Subcutaneous and abdominal fat were collected to analyse fatty acid composition. The alternative diet had no negative effect on ducks’ growth performance parameters and dressing percentage. The replacement of SBM with yellow lupin and rapeseed meal increased n-3 fatty acid content, which is important for consumers. In conclusion, SBM can be replaced with feed containing 60.10% of yellow lupin and 14.00% of rapeseed meal in concentrate. These sources of protein are mainly recommended for small poultry farms, which do not always have access to SBM and prepare poultry feed from their own crops.

## Introduction

Pekin ducks are one of the most popular breeds of waterfowl in the world kept for meat^1^. They are characterized by high growth performance, and there are different strategies for rearing these ducks^2,3^. Growth performance and the quality of meat largely depend on diet and the choice of appropriate feed components^4^. Diet also influences the content of fatty acids in meat and fat, which is important in the context of consumers’ health^5^. Generally, SBM is the main source of protein in poultry diets, but it is usually obtained from genetically modified plants and its use is now raising concerns among consumers^6^. Studies have not provided compelling evidence on the negative effects of animal diets based on SBM^7^. Nevertheless, increasing soybean prices prompted researchers to investigate other sources of protein suitable for animal diets^8^. Biesek et al.^9^ concluded that legumes, particularly lupin seeds, are a promising source of protein for poultry diets, although they do not provide sufficient amounts of starch or easily digestible carbohydrates. In the past, the use of legume seeds was limited because of their high content of antinutrients, despite the fact that lupins, depending on the cultivar, contain approx. 40% of protein. However, new varieties of lupin created in breeding studies are characterised by much lower levels of antinutrients deteriorating growth performance in poultry^10–12^. Another alternative source of energy and protein is rapeseed meal (RSM)^7^, a by-product of rape processing^13^. RSM is rich in protein (34%), and is easily available for the production of animal feed, but it also contains antinutrient glucosinolates and therefore cannot replace SBM completely^14^. Hang et al.^13^ concluded that the addition of vitamins to feed with a 5 or 10% inclusion of RSM can minimize the negative effects of antinutrients. Kuźniacka et al.^12^ compared the effects of diets containing different sources of protein on the quality of meat from ducks. The best quality parameters were found in ducks fed diets with 57.78–42.22% inclusions of yellow lupin and 17.87–34.49% inclusions of RSM in starter and grower feeds, respectively. Similar results were reported by Banaszak et al.^15^, who proposed a 68.98% inclusion of yellow lupin in feed concentrate for ducks. The researchers concluded that the proposed feed ration can partly substitute SBM, since it had no negative effect on most quality traits of meat, while yellow lupin had a positive effect on fatty acid composition in breast muscles from ducks.

Tested hypothesis: A diet with a 60.10% inclusion of yellow lupin and a 14.00% inclusion of rapeseed meal as substitutes for SBM influences growth performance parameters, meat quality and fatty acid composition in fat from ducks.

The aim of this study was to compare growth performance, quality of carcass and meat, and fatty acid composition in subcutaneous and abdominal fat from ducks fed a diet based on yellow lupin and rapeseed meal used as sources of protein alternative to SBM.

## Results

### Growth performance

**Figure 1:**
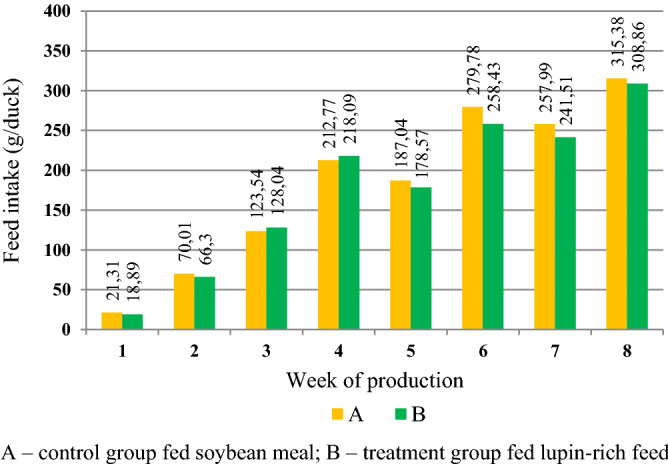
Mean daily feed intake (g/duck) during growth.

Growth performance parameters are presented in Table 1. Data were not analysed with statistical methods. Mortality of ducks differed between dietary treatment groups and sexes. The highest mortality was found in males from group B (7.81%), and the lowest in males from group A (1.56%). All cases of mortality were recorded at the early rearing period (weak ducklings). Feed conversion ratio (FCR) in all groups ranged from 3.11 to 3.30 kg/kg. Feed intake (FI) was from 10.49 kg in males from group A to 9.86 kg in females from group B. The mean daily feed intake is presented in Fig. 1. For each week of production; no significant differences were noted. Daily feed intake was 18.89–21.31 g in week 1 and 308.86–315.38 g in week 8. Body weight of ducks (Table 2) in weeks 2–8 differed between treatment groups (*P* = 0.000), and was significantly higher in group A. Body weight in weeks 5 and 8 was significantly higher in male ducks (*P* = 0.000). There was also an interaction between performance variables in weeks 1 to 5 (*P* = 0.001; *P* = 0.000). Despite differences in body weight in subsequent weeks of production between dietary treatments and sex of ducks, body weight gain (BWG) in treatment groups at the end of the rearing period was comparable (*P* > 0.05). BWG was significantly higher in male ducks than in females (*P* = 0.000). Significant differences between groups A and B were found in weeks 2, 3, 5 and 6 of production (BWG was higher in group A), while differences between sexes were found in the same weeks as for treatment groups, except week 8. There was also an interaction effect between parameters recorded in weeks 1, 2, 4 and 6 (Table 3). The analysis of data in Table 4 presenting zoometric traits and body conformation indices of ducks revealed sex-associated differences: body length, wing length, compactness index and long-leggedness index were significantly higher in males than in females. A significant interaction between variables analysed in the experiment was found for chest circumference and massiveness index (*P* = 0.032; *P* = 0.002).

**Table 1:** Growth performance parameters in ducks.

Group	Sex	Mortality (%)	FCR per kg of body weight gain (kg/kg)	FI—feed intake per duck during growth (kg)	European Broiler Index (EBI)
A	♂♂	1.56	3.11	10.49	192.18
	♀♀	3.28	3.13	10.00	177.13
B	♂♂	7.81	3.21	10.06	158.14
	♀♀	4.84	3.30	9.86	156.02

*A* ducks fed a diet based on soybean meal; *B* ducks fed a diet based on yellow lupin and rapeseed meal; ♂♂Males, ♀♀Females.

**Table 2:** Means (g) and coefficients of variation (%) for body weight in subsequent weeks of duck growth (${\overline{\text{x}}}$, V) (number of ducks: N = 102 per group).

Age (week)	Group		V (%)	Sex		*P* value		
	A	B		♂♂	♀♀	Group	Sex	Interaction
0	60.59	61.22	7.64	61.15	60.66	0.295	0.420	0.345
1	140.40	140.08	14.52	139.69	140.78	0.906	0.688	0.000^x^
2	454.33^a^	399.64^b^	19.11	417.44	436.97	0.000	0.078	0.000^x^
3	830.92^a^	719.14^b^	16.93	777.93	773.59	0.000	0.737	0.001^x^
4	1,411.34^a^	1,314.96^b^	15.13	1,359.21	1,368.19	0.000	0.771	0.001^x^
5	1901.13^a^	1741.66^b^	13.98	1786.05^b^	1857.84^a^	0.000	0.041	0.000^x^
6	2,571.75^a^	2,327.86^b^	11.72	2,456.27	2,446.53	0.000	0.731	0.145
7	3,005.39^a^	2,788.42^b^	10.25	2,922.00	2,875.00	0.000	0.196	0.829
8	3,307.63^a^	3,096.28^b^	9.29	3,285.94^a^	3,122.67^b^	0.000	0.000	0.526

*A* ducks fed an SBM-based diet; *B* ducks fed a diet based on yellow lupin and rapeseed meal.

^a,b^Means in rows marked with different letters differ significantly between groups (*P* ≤ 0.05).

^x^Significant group: sex interaction (*P* ≤ 0.05).

**Table 3:** Body weight gain (g) in subsequent weeks of duck growth (${\overline{\text{x}}}$) (N = 102 ducks per group).

Age (week)	Group		Sex		*P* value		
	A	B	Males	Females	Group	Sex	Interaction
1	79.81	78.86	78.53	80.12	0.702	0.528	0.000^x^
2	313.93^a^	259.56^b^	277.75^b^	296.19^a^	0.000	0.043	0.003^x^
3	376.60^a^	319.50^b^	360.49^a^	336.63^b^	0.000	0.009	0.103
4	580.42	595.83	581.28	594.60	0.295	0.358	0.017^x^
5	489.79^a^	426.70^b^	426.84^b^	489.65^a^	0.004	0.005	0.118
6	670.62^a^	583.53^b^	667.55^a^	588.69^b^	0.007	0.013	0.031^x^
7	433.64	460.55	465.85	428.47	0.253	0.116	0.051
8	302.24	307.86	363.82^a^	247.67^b^	0.806	0.000	0.153

*A* ducks fed an SBM-based diet; *B* ducks fed a diet based on yellow lupin and rapeseed meal.

^a,b^Means in rows marked with different letters differ significantly (*P* ≤ 0.05).

^x^Significant group: sex interaction (*P* ≤ 0.05).

**Table 4:** Zoometric traits (cm) and body conformation indices (%) of ducks (${\overline{\text{x}}}$ ± SD) (N = 10 ducks per group).

Parameter	Group		SD	Sex		*P* value		
	A	B		Males	Females	Group	Sex	Interaction
Body length (cm)	40.55	40.55	1.54	42.30^a^	38.80^b^	0.085	0.000	1.000
Abdomen length (cm)	23.55	23.45	1.16	24.00	23.00	0.854	0.081	0.584
Sternum length (cm)	17.50	17.20	0.75	17.15	17.55	0.384	0.250	0.250
Shank length (cm)	5.27	5.25	0.42	5.24	5.28	0.920	0.842	0.205
Wing length (cm)	10.50	10.30	0.27	10.80^a^	10.00^b^	0.177	0.000	0.121
Chest circumference (cm)	35.60	34.95	1.39	35.45	35.10	0.059	0.291	0.032^x^
Massiveness index (%)	13.41	14.00	0.74	13.59	13.89	0.070	0.391	0.002^x^
Compactness index (%)	151.64	149.34	7.74	146.49^b^	154.50^a^	0.533	0.041	0.800
Long-leggedness index (%)	13.06	12.98	1.26	12.41^b^	13.36^a^	0.889	0.050	0.763

*A* ducks fed an SBM-based diet; *B* ducks fed a diet based on yellow lupin and rapeseed meal.

^a,b^Means in rows marked with different letters differ significantly (*P* ≤ 0.05).

^x^Significant group: sex interaction (*P* ≤ 0.05).

### Carcass traits and physicochemical properties of meat

Live body weight of ducks selected for slaughter was significantly higher in group A than in group B, and higher in males than in females. Differences were significant at *P* = 0.000 for both analysed variables. There were significant differences in the chilled carcass weight between dietary treatment groups (higher carcass weight in group A than in B, *P* = 0.000). Dressing percentage was significantly lower in males than in females (*P* = 0.008). The weight of breast muscles and wings was significantly higher in group A fed an SBM-based diet (*P* < 0.05). The proportion of skin with subcutaneous fat in carcass was significantly higher in group B compared to group A (*P* = 0.028). Significant sex-related differences were found for the weight of skin with subcutaneous fat, abdominal fat, weight of stomach and liver, and the proportion of skin with subcutaneous fat, abdominal fat and the proportion of neck in the carcass; all these parameters except weight of stomach and liver were higher in females (*P* < 0.05). No significant differences were found between other variables (*P* > 0.05). There was no significant interaction between variables: treatment group and sex (P > 0.05), except for live body weight of ducks (*P* = 0.000) (Table 5). Better cooking loss of breast muscles was found in birds from group B fed a diet based on yellow lupin compared to group A (*P* = 0.026). Other physicochemical parameters did not differ significantly between groups for both variables (*P* > 0.05). No interaction effect was found between treatment group and sex (*P* > 0.05) (Table 6). Higher content of linolenic acid (C18:3 n-3) and higher content of eicosenoic acid (C20:1 n-9) in subcutaneous fat in group B compared to control ducks fed the SBM-based diet was found. The percentage of n-3 fatty acids (C18:n-3) was significantly higher in group B compared to group A, but the ratio PUFA n-6/n-3 was significantly lower (*P* = 0.000). Fatty acid composition was also analysed in relation to sex. The content of pentadecanoic (C15:0), palmitoleic (C16:1), heptadecanoic (C17:0), linoleic acid (C18:2 n-6), and polyunsaturated acids (PUFA), including omega-6 and hypocholesterolemic FA (DFA), as well as PUFA/SFA and DFA/OFA (hypercholesterolemic fatty acids) were significantly higher in males than in females (*P* < 0.05) (Table 7). No interaction effect between variables was found (*P* > 0.05). The content of specific fatty acids was also lower in abdominal fat from control group A compared to group B. There were significant differences in the content of linolenic acid (C18:3 n-3), eicosenoic acid (C20:1 n-9) and docosanoic acid (C22:0), and abdominal fat from group A was characterised by a lower content of n-3 acids, but a higher n-6/n-3 ratio (*P* < 0.05). There were also differences in fatty acid composition, including higher content of heptadecanoic (C17:0), linoleic (C18:2 n-6), docosanoic (C22:0) acids, total PUFA, and the PUFA/SFA ratio in males than in females. The content of palmitoleic acid (C16:1) was higher in females (*P* = 0.045) (Table 8). No interaction effect between the diet-sex was found (*P* > 0.05).

**Table 5:** Means and coefficients of variation (%) for body weight and carcass weight, dressing percentage and weight of carcass elements and percentage share of cuts in carcasses (${\overline{\text{x}}}$, V) (N = 10 ducks per group).

Parameter		Group		V (%)	Sex		*P* value		
		A	B		Males	Females	Group	Sex	Interaction
**Weight**									
g	Body, before slaughter	3,343.30^a^	3,096.00^b^	1.00	3,295.90^a^	3,141.40^b^	0.000	0.000	0.000^x^
g	Chilled carcass	2,312.10^a^	2,144.63^b^	2.99	2,237.61	2,219.12	0.000	0.580	0.723
%	Dressing percentage	69.90	70.12	3.00	68.55^b^	71.48^a^	0.822	0.008	0.320
**Weight and proportion in carcass**									
g	Breast muscles	452.67^a^	388.45^b^	10.26	412.30	428.82	0.004	0.402	0.633
%		19.59	18.11	9.68	18.38	19.31	0.090	0.275	0.680
g	Leg muscles	295.18	284.02	6.55	297.82	281.38	0.217	0.077	0.342
%		12.76	13.24	5.37	13.31	12.69	0.143	0.070	0.365
g	Wings	276.23^a^	262.57^b^	5.06	275.22	263.58	0.045	0.082	0.271
%		11.96	12.25	5.84	12.32	11.89	0.402	0.226	0.501
g	Skin with subcutaneous fat	382.84	397.88	10.43	366.74^b^	413.98^a^	0.430	0.022	0.890
%		16.58^b^	18.55^a^	10.21	16.42^b^	18.71^a^	0.028	0.013	0.891
g	Abdominal fat	24.04	25.84	21.24	21.55^b^	28.33^a^	0.485	0.016	0.919
%		1.00	1.21	24.30	0.92^b^	1.28^a^	0.120	0.011	0.857
g	Skin with neck	117.62	103.29	14.59	115.45	105.46	0.065	0.186	0.109
%		5.09	4.82	14.62	5.17	4.73	0.413	0.197	0.088
g	Neck	160.87	160.51	7.16	166.14	155.24	0.950	0.074	0.343
%		7.40	7.49	9.80	7.88^a^	7.01^b^	0.820	0.043	0.772
g	Carcass remains	558.52	509.72	9.68	558.10	510.14	0.052	0.055	0.519
%		24.16	23.76	9.37	24.93	23.00	0.704	0.080	0.668
g	Stomach	105.78	100.85	9.65	110.17^a^	96.46^b^	0.306	0.010	0.744
g	Liver	71.95	75.55	13.08	80.70^a^	66.80^b^	0.421	0.006	0.214
g	Heart	17.61	16.74	9.03	17.64	16.71	0.259	0.232	0.326

*A* ducks fed an SBM-based diet; *B* ducks fed a diet based on yellow lupin and rapeseed meal.

^a,b^Means in rows marked with different letters differ significantly (*P* ≤ 0.05).

^x^Significant group: sex interaction (*P* ≤ 0.05).

**Table 6:** Physicochemical parameters of breast and leg muscles from ducks (N = 10 ducks per group).

Parameter	Element	Group		SD	Sex		*P* value		
		A	B		Males	Females	Group	Sex	Interaction
pH_24_	Breast	5.83	5.82	0.06	5.81	5.84	0.670	0.347	0.768
Water-holding capacity (%)	Breast	58.05	60.32	9.87	60.94	58.01	0.165	0.104	0.056
	Legs	64.78	62.82	7.71	62.93	64.65	0.122	0.177	0.318
Drip loss (%)	Breast	1.47	1.37	0.45	1.39	1.45	0.643	0.780	0.762
	Legs	1.26	0.71	0.68	0.90	1.08	0.106	0.588	0.812
Cooking loss (%)	Breast	25.12^a^	22.22^b^	2.33	24.66	22.68	0.026	0.114	0.709
	Legs	24.01	25.00	2.82	24.45	24.63	0.485	0.893	0.289
L*—colour lightness	Skin	65.87	65.22	2.21	65.56	65.53	0.554	0.973	0.326
	Breast muscles	33.99	34.98	1.72	34.70	34.26	0.227	0.587	0.652
	Leg muscles	35.10	37.30	2.92	36.12	36.28	0.123	0.907	0.552
a*-colour redness	Skin	4.66	4.57	1.21	4.32	4.91	0.867	0.299	0.102
	Breast muscles	14.60	14.58	1.03	14.87	14.31	0.972	0.281	0.244
	Leg muscles	13.76	13.80	1.44	14.35	13.20	0.952	0.110	0.417
b*-colour yellowness	Skin	12.57	13.68	2.04	13.28	12.97	0.244	0.734	0.397
	Breast muscles	2.06	1.43	1.61	1.85	1.63	0.341	0.740	0.950
	Leg muscles	3.45	4.19	1.35	3.97	3.66	0.265	0.634	0.441

*A* ducks fed an SBM-based diet; *B* ducks fed a diet based on yellow lupin and rapeseed meal.

^a.b^Means in rows marked with different letters differ significantly (*P* ≤ 0.05).

**Table 7:** Fatty acid composition (% of total fatty acids) in subcutaneous fat from ducks (${\overline{\text{x}}}$ ± SD) (N = 10 ducks per group).

Fatty acids	Group		SD	Sex		*P* value		
	A	B		Males	Females	Group	Sex	Interaction
C14:0	1.10	1.07	0.10	1.08	1.09	0.434	0.946	0.339
C15:0	0.11	0.12	0.02	0.12^a^	0.11^b^	0.064	0.018	0.335
C16:0	47.06	46.34	1.29	46.10	47.25	0.206	0.059	0.887
C16:1	2.09	2.19	0.31	1.98^b^	2.31^a^	0.432	0.016	0.879
C17:0	0.14	0.16	0.03	0.16^a^	0.13^b^	0.086	0.006	0.925
C18:0	11.09	10.90	1.38	11.30	10.69	0.767	0.359	0.741
C18:1 n-9	29.51	29.82	2.24	29.53	29.79	0.784	0.817	0.736
C18:2 n-6	8.02	8.36	0.81	8.66^a^	7.72^b^	0.270	0.007	0.201
C18:3 n-3	0.60^b^	0.70^a^	0.08	0.69	0.62	0.006	0.059	0.284
C20:1 n-9	0.19^b^	0.22^a^	0.02	0.21	0.20	0.002	0.498	0.369
C22:0	0.09^b^	0.13^a^	0.03	0.12	0.10	0.011	0.053	0.939
SFA	59.59	58.71	2.07	58.93	59.36	0.382	0.667	0.928
UFA	40.41	41.30	2.07	41.07	40.64	0.379	0.668	0.927
MUFA	31.79	32.23	2.33	31.72	32.30	0.715	0.628	0.770
PUFA	8.62	9.07	0.68	9.35^a^	8.34^b^	0.195	0.008	0.202
n-3	0.60^b^	0.70^a^	0.07	0.69	0.62	0.006	0.059	0.284
n-6	8.02	8.36	0.62	8.66^a^	7.72^b^	0.270	0.007	0.201
n-9	29.70	30.04	2.19	29.74	30.00	0.763	0.822	0.743
DFA	49.41	50.00	1.28	50.39^a^	49.02^b^	0.328	0.034	0.810
OFA	48.16	47.40	1.26	47.23	48.34	0.205	0.069	0.834
UFA/SFA	0.68	0.70	0.06	0.54	0.55	0.397	0.630	0.887
MUFA/SFA	0.54	0.55	0.06	0.54	0.55	0.628	0.869	0.910
PUFA/SFA	0.14	0.15	0.01	0.16^a^	0.14^b^	0.055	0.002	0.158
DFA/SFA	0.83	0.85	0.05	0.86	0.83	0.330	0.181	0.986
DFA/OFA	1.03	1.06	0.05	1.07^a^	1.02^b^	0.251	0.047	0.817
n-6/ n-3	13.35^a^	11.96^b^	0.43	12.72	12.59	0.000	0.691	0.728
n-9/ n-6	3.78	3.61	0.41	3.48	3.91	0.476	0.069	0.405
n-9/ n-3	50.39	43.37	6.79	44.21	49.55	0.058	0.140	0.391

*A* ducks fed an SBM-based diet; *B* ducks fed a diet based on yellow lupin and rapeseed meal.

*DFA* hypocholesterolemic fatty acids, *OFA* hypercholesterolemic fatty acids, *UFA* unsaturated fatty acids, *SFA* saturated fatty acids, *MUFA* monounsaturated fatty acids, *PUFA* polyunsaturated fatty acids.

^a,b^Means in rows marked with different letters differ significantly (*P* ≤ 0.05).

**Table 8:** Fatty acid composition (% of total fatty acids) in abdominal fat from ducks (${\overline{\text{x}}}$ ± SD) (N = 10 ducks per group).

Fatty acids	Group		SD	Sex		*P* value		
	A	B		Males	Females	Group	Sex	Interaction
C14:0	1.15	1.10	0.10	1.12	1.13	0.318	0.704	0.231
C15:0	0.11	0.12	0.02	0.13	0.11	0.226	0.051	0.461
C16:0	46.20	46.17	1.19	45.64	46.68	0.889	0.065	0.756
C16:1	1.95	2.04	0.33	1.82^b^	2.14^a^	0.593	0.045	0.835
C17:0	0.15	0.16	0.03	0.17^a^	0.14^b^	0.294	0.014	0.548
C18:0	12.38	11.54	1.39	12.26	11.73	0.278	0.545	0.678
C18:1 n-9	28.87	29.40	2.36	29.03	29.20	0.649	0.935	0.578
C18:2 n-6	8.33	8.46	0.74	8.86^a^	7.97^b^	0.593	0.007	0.128
C18:3 n-3	0.59^b^	0.67^a^	0.07	0.65	0.61	0.025	0.187	0.137
C20:1 n-9	0.20^b^	0.23^a^	0.02	0.22	0.21	0.000	0.099	0.841
C22:0	0.08^b^	0.11^a^	0.04	0.11^a^	0.08^b^	0.024	0.043	0.680
SFA	60.07	59.21	2.04	59.42	59.88	0.453	0.658	0.843
UFA	39.93	40.80	2.05	40.58	40.13	0.452	0.661	0.844
MUFA	31.08	31.78	2.38	31.18	31.63	0.606	0.758	0.597
PUFA	8.92	9.12	0.60	9.51^a^	8.57^b^	0.513	0.007	0.121
n-3	0.59^b^	0.67^a^	0.06	0.65	0.61	0.024	0.160	0.137
n-6	8.33	8.46	0.57	8.86^a^	7.97^b^	0.649	0.006	0.128
n-9	29.06	29.63	2.22	29.25	29.41	0.647	0.916	0.580
DFA	52.31	52.33	1.17	52.83	51.86	0.969	0.101	0.877
OFA	47.35	47.28	1.15	46.76	47.81	0.901	0.072	0.850
UFA/SFA	0.67	0.69	0.06	0.69	0.67	0.473	0.570	0.899
MUFA/SFA	0.52	0.54	0.06	0.53	0.53	0.583	0.983	0.715
PUFA/SFA	0.15	0.15	0.01	0.16^a^	0.14^b^	0.191	0.000	0.083
DFA/SFA	0.87	0.88	0.05	0.89	0.87	0.639	0.287	0.997
DFA/OFA	1.11	1.11	0.05	1.13	1.09	0.942	0.078	0.846
n-6/3	14.08^a^	12.75^b^	0.73	13.76	13.17	0.004	0.174	0.510
n-9/6	3.55	3.52	0.43	3.34	3.71	0.911	0.118	0.295
n-9/3	49.76	44.95	6.49	45.78	49.02	0.157	0.293	0.174

*A* ducks fed an SBM-based diet; *B* ducks fed a diet based on yellow lupin and rapeseed meal.

*DFA* hypocholesterolemic fatty acids, *OFA* hypercholesterolemic fatty acids, *UFA* unsaturated fatty acids, *SFA* saturated fatty acids, *MUFA* monounsaturated fatty acids, *PUFA* polyunsaturated fatty acids; n-3 OMEGA 3, n-6 OMEGA 6, n-9 OMEGA 9.

^a b^Means in rows marked with different letters differ significantly (*P* ≤ 0.05).

## Discussion

### Growth performance

In our study the European broiler index (EBI) for ducks ranged from 156.02 (B) to 192.18 (A), and FCR from 3.11 (♂♂ A) to 3.21 (♂♂ B). As Wężyk et al.^16^ reported good EBI value is approximately 190. Total feed intake over the 8-week rearing period was approx. 10 kg. Feed intake in control and treatment groups was comparable. Zduńczyk et al.^17^ reported that the inclusion of yellow lupin seeds (LL_8, 16 and 24_) in the diet of turkeys had no negative effect on feed intake. Hejdysz et al.^18^ also demonstrated no significant effect of narrow-leaved lupin and type of processed seeds on feed intake and FCR in broiler chicken. In our study the mortality rate of ducks was from 1.56% (♂♂ A) to 7.81% (♂♂ B). Presumably, diet was one of the main factors determining the survival of birds. In our study the body weight and weekly body weight gains were lower in ducks from the treatment group compared to control birds (*P* ≤ 0.05). Kuźniacka et al.^19^ reported that the final body weight of ducks fed diets with the inclusion of lupin seeds was comparable to the body weight of ducks fed a balanced SBM-based feed. Biesiada-Drzazga et al.^20^ found higher body weight and growth rate in Pekin ducks (♂♀) at 14 and 28 days-old, but lower at 49 days-old compared to the body weight of ducks measured in our study. Mierlita and Popovici^21^ reported from their experimental study on Ross 308 broiler chickens the significantly highest body weight in 21-day-old birds fed a standard SBM-based diet and the lowest in the LA_40_ group. However, the body weight of 35-day-old broiler chickens was comparable. A 40 and 60% inclusion of lupin flour per feed ration had no adverse effect on the body weight of birds measured at the end of the rearing period (day 42). Daily feed intake in control and treatment groups was comparable. Suchỳ et al.^22^ also found no significant differences between the body weight of Ross 308 broiler chickens fed white lupin (LA) seeds LA_6.66–31.03_ measured on days 15, 30 and 42 of rearing. In week 2 of life, the highest body weight gain was found in ducks from the control group A (104.39%) and treatment group B (95.37%). The values of body conformation indices, sternum crest length and chest circumference were higher in female ducks. Experiments conducted by Kokoszyński and Bernacki^23^ conducted on P44 and P55 ducks revealed lower values compared to those found in our study.

### Carcass traits and physicochemical properties of meat

Body weight and carcass weight were significantly higher in control ducks than in ducks from the treatment group. The analysis of carcass quality revealed significant differences in the weight of breast muscles and wings, as well as the proportion of wings in gutted carcass with neck. Kuźniacka et al.^19^ reported a slightly higher dressing percentage in ducks fed a balanced feed based on narrow lupin seeds compared to ducks fed mixtures with the inclusion of soybean meal. An experimental study by Witak et al.^24^ revealed that the inclusion of yellow lupin seeds in the diet of A44 ducks (2.5, 5 and 10% in weeks 1 to 3 of life, and 7.5, 10 and 15% from week 4 of rearing to slaughter) had no effect on the weight of gutted carcass with neck (g) and the weight and proportion of skin with subcutaneous fat, abdominal fat, breast muscles and leg muscles, or carcass remains. Witak et al.^24^ reported a negative (*P* ≤ 0.05) effect of a 10% inclusion of lupin seeds on the dressing percentage of ducks compared to the control group and other treatment groups. There were also significant sex-related differences in the dressing percentage and the proportion of subcutaneous fat in carcass, which were higher in female ducks, similar to our study. Only the weight of carcass remains was higher in male ducks. The proportion of individual elements of carcass reported by Witak et al.^24^ was slightly higher than in our study. In the study by Suchỳ et al.^22^ carcass weight and the weight and proportion of breast muscles in carcass was significantly lower in Ross 308 broiler chickens fed a diet with the inclusion of white lupin seeds (LA) compared to the control group (SBM), but the dressing percentage of birds was comparable (70.99% in the control group vs. 73.69% in treatment groups). Diet also had no negative effect on the weight and proportion of neck and legs in carcass. Comparable results were found in our study. Our study found no significant effect of the source of protein in the diet on the weight of carcass elements, but the weight of stomach and liver was significantly higher in males compared to females. In our study the proportion of wings in the total weight of carcass was between 11.89% for female ducks and 12.32% for male ducks. Similar data on the weight of duck wings were reported by Kokoszyński et al.^25^.

In our study, dietary treatments had no significant effect on the colour of duck breast and leg muscles. Our findings were different from those reported by other researchers^26–28^. Other studies^29^ found that muscles from turkeys fed LL_16_ and LL_24_ diets were characterised by a significantly greater yellowness (b*) compared to control birds. An experimental study by Witak et al.^24^ found no significant differences in the colour of breast and leg muscles between the control ducks receiving a feed mixture with the addition of SBM, and treatment groups (LL_2.5–15_) or between males and females, which is consistent with our results.

Moreover, Witak et al.^24^ reported that the source of plant protein did not influence the water-holding capacity of breast and leg muscles from broiler ducks. The sex of birds was the only variable with a significant impact on the water-holding capacity of breast muscles and was higher in female than in male birds. On the other hand, Kuźniacka et al.^19^ reported higher water-holding capacity of breast and leg muscles from ducks fed a diet with the inclusion of lupin seeds compared to birds receiving a feed mixture with SBM. Wu et al.^30^ reported that in Cherry Valley ducks cooking loss was 38.10%, and drip loss was 7.48%. In our study we found no relationship between the sex of ducks or their diet and the pH of breast muscles, which according to literature data is in the range of 5.65–6.20 and depends on the level of glycogen reserves in muscles^27,31,32^. The value of pH decreases post-mortem due to the increased concentration of lactic acid in muscles, and this process determines meat quality. The rapid drop in pH may result in meat paleness, reduced water-holding capacity and an excessively soft structure of meat^33^. In our experiments the post-mortem drop in the pH of breast muscles was normal, which indicates normal metabolic changes in muscles^28^. Krawczyk et al.^29^ did not observe any significant differences in the pH of turkey meat measured 24 h post-mortem between the control group (SBM) and treatment groups (LL_8–24_). The pH_24_ of breast muscles from ducks measured in our study was higher than 5.8. Witak et al.^24^ reported slightly lower pH values for breast muscles (5.74 to 5.78) from ducks on an LL diet compared to our findings. Wu et al.^30^ reported that the pH of muscles from Cherry Valley ducks was in the range of 5.97 (pH_45 min_) to 5.77 (pH_24h_). The water-holding capacity of duck muscles was comparable between groups and sexes of birds.

### Lipid content and fatty acid composition of tissues

Fat from lupin seeds is characterised by a high content of unsaturated fatty acids, especially linoleic acid (C18:2 n-6)^34^. Mieczkowska and Smulikowska^35^ reported that the dietary inclusion of lupin seeds influenced the level of oleic acid (C18:1 n-9) and α-linolenic acid (C18:3 n-3) in fat from broilers. Diet has a modifying effect on fatty acid composition in somatic lipids, and this improves the nutritional properties of poultry meat. Results of the cited studies correspond with our findings, since we measured significantly higher content of α-linolenic acid in subcutaneous and abdominal fat from ducks on a diet with the inclusion of lupin seeds. Biesiada-Drzazga^31^ indicated that the partial replacement of soybean meal with lupin seed meal and rapeseed meal in the diet of White Kołuda geese reared for 10 weeks had a positive effect and was associated with a 2.2% reduction in the total content of SFA, and a 2.85% increase in the total content of MUFA. On the other hand, a significantly lower content of SFA and higher content of UFA was found in skin with subcutaneous fat (by 1.7% on average) and in leg muscles (by 1.2 and 1.7%, respectively) from geese receiving sunflower meal or lupin meal with sunflower meal^32^. Another study by Biesiada-Drzazga^36^ revealed no differences in fatty acid composition for breast muscle, leg muscles and skin with subcutaneous fat between geese fed soybean meal and geese fed a diet with an admixture of rapeseed meal. Skin with subcutaneous fat was characterised by a more beneficial fatty acid composition compared to abdominal fat (higher UFA/SFA ratio), which corresponds with findings by Karpińska and Batura^37^. In oat geese, a dietary inclusion of sunflower seeds (5% in weeks 0 to 3, 9% in weeks 4 to 8 and 14% in weeks 9 to 10) had a positive effect on the content of oleic acid (C18:1 n-9) in abdominal fat, subcutaneous fat and lipids from breast muscles, and on the content of palmitoleic acid (C16:1) in subcutaneous fat and breast muscles^38^. Feeding turkeys with a mixture based on yellow lupin seeds (LL_8–24_) influenced fatty acid composition in breast muscles by reducing the level of saturated fatty acids (SFA), including palmitic (C16:0) and myristic acid (C14:0), and increasing the levels of polyunsaturated fatty acids (PUFA): linoleic (C18:2 n-6) and linolenic acid (C18:3 n-3)^29^. Experimental studies by Kiczorowska et al.^39^ revealed significantly higher levels of palmitoleic (C16:1) and arachidonic acid (C20:4 n-9), and a significantly lower level of myristic acid (C14:0) in abdominal fat from broiler chickens fed with a 50% inclusion of raw peas. This diet was also associated with increased content of palmitic (C16:0) and γ-linolenic acid (C18:3 n-6) in lipids from leg muscles compared to controls fed SBM.

In conclusion, yellow lupin in combination with rapeseed meal can be a source of protein in feed for broiler ducks, as shown by growth performance results and the dressing percentage of the carcass. Growth performance parameters and dressing percentage achieved for ducks fed this diet were comparable to those in birds on the balanced SBM-based diet. The use of balanced feeds with the inclusion of yellow lupin and rapeseed meal has a positive effect on fatty acid composition in abdominal and subcutaneous fat from broiler ducks. Meat from ducks fed this type of diet was characterized by a highest level of hypocholesterolemic and PUFA n-3. Feed with the inclusion of lupin seeds and rapeseed meal can be used in the diet of ducks at small poultry farms operating semi-intensive production, as indicated by the good quality of obtained meat. It is important for small farms, because not everybody has a possibility for own crops of soybean meal. In aspect of consumer market, nowadays is niche of non-GMO products, so it could give a wider choice for potential consumers.

## Materials and methods

The research were done with recommendations of directive no. 2010/63/EU and resolution 13/2016 of the National Ethics Committee for Animal Experiments of June 17, 2016. The approval of Ethic Committee was not required. The slaughter of birds was carried out in accordance with the applicable rules on the handling of animals at the time of slaughter, including humane treatment. Also the methods used in the meat quality tests were carried out in accordance with the current and commonly used methods.

### Animals and diets

The study was conducted on 204 Cherry Valley English Pekin ducks. Each duckling was weighted on Radwag PS 750/X scales, sexed, and marked with a jiffy wing band. Ducklings were reared in closed facilities under controlled environmental conditions (in accordance with Polish law, ducks were kept to a maximum of 10.5 kg/m^2^). Air temperature was 27 to 31 °C in week 1, 23 to 29 °C in week 2, and 23 to 26 °C in week 3. The mean relative air humidity was 65%. From the third week of life birds had access to outdoor pens. Birds were divided into two groups (N = 102), each group was in five repetition, and subgroups depending on sex, with 51 males and 51 females in each subgroup. Control birds (A) received a mixture with concentrate containing soybean meal (SBM), and the treatment group (B) received a balanced feed with lupin seeds. The diet of ducks (A, B) was a mixture of 40% of concentrate and 60% of wheat from week 1 to 3 of life, and then 30% of concentrate and 70% of wheat from week 4 to 8. The components of the concentrate and the nutritional value of feed mixture are presented in Table 9. The chemical composition of yellow lupin seeds, cv. Mister, is presented in Table 10. The chemical composition of rapeseed meal was not presented since it was not the main objective of the study. To supplement nutrients in each treatment group, between weeks 2 and 8 birds received powder product containing amino acids and vitamins (dose 125 g/1,000 L drinking water, Amino-Vitasol WSP, Medivet), as well as SELVITA Vitamin E + Selenium (INVESA) at a dose of 1,000 ml/ 4,000 l drinking water between weeks 5 and 8. Supplementation was the same in all groups.

**Table 9:** Components of concentrates for ducks.

Component (%)	A	B
Wheat	9.00	17.50
Soybean meal (Hipro)	57.90	–
Yellow lupin (Mister)	–	60.10
Rapeseed meal	–	14.00
Wheat bran	25.00	–
Monocalcium phosphate	2.70	2.90
Fodder chalk	2.50	2.30
Fodder salt	0.50	0.60
Sodium carbonate	0.40	0.40
L-lysine/technically pure/	–	0.10
DL-methionine	0.50	0.60
Premix grower 5%, *	1.50	1.50
Calcium	2.24	2.20
Available phosphorus	0.58	0.58
Lysine	1.90	1.90
Methionine	0.90	0.90
Cysteine	0.81	0.79
Threonine	0.81	0.82
Tryptophane	3.24	3.30
Nutritional value per kg of complete feed		
Metabolizable energy (MJ)	11.41	11.41
Metabolizable energy (kcal)	2,725.23	2,725.23
Crude protein (%)	17.30	17.30

*Vitamin–mineral premix provided per kg diet: Mn, 55 mg; Zn, 50 mg; Fe, 80 mg; Cu, 5 mg; Se, 0.1 mg; I, 0.36 mg; Na, 1.6 g, retinol, 2.48 mg; cholecalciferol 25 μg; DL-ɑ-tocopherol, 60 mg; cyanocobalamin, 0.012 mg; menadione sodium bisulphite, 1.1 mg; niacin, 53 mg; choline chloride, 1,020 mg; folic acid, 0.75 mg; biotin, 0.25 mg; riboflavin, 5.5 mg.

**Table 10:** Chemical composition of yellow lupin seeds, cultivar Mister.

Parameter	Unit	Yellow lupin, cv. Mister
Dry matter	%	89.01
Crude ash	%	4.15
Crude protein	%	38.98
Crude fibre	%	19.23
ADF	%	24.24
NDF	%	28.24
Crude fat	%	5.26
Starch	%	–
Energy	MJ/kg	20.49
	kcal/kg	4,893.95
Viscose	cP	1.09
Asp	%	8.81
Thr	%	3.17
Ser	%	4.24
Glut	%	24.46
Pro	%	6.08
Gly	%	3.47
Ala	%	2.83
Val	%	3.17
Iso	%	3.20
Leu	%	6.50
Tyr	%	3.24
Phe	%	4.24
His	%	3.32
Lys	%	4.76
Arg	%	10.12
Total amino acids:	%	39.29
Ca	g/kg DM	2.95
K	g/kg DM	12.66
P	g/kg DM	7.47
Na	g/kg DM	0.08
Mg	g/kg DM	3.14
Mn	g/kg DM	0.08
Cu	g/kg DM	0.02
Fe	g/kg DM	0.13
Zn	g/kg DM	0.07
Total alkaloids:	mg/kg	270
Angustifoline	%	–
Isolupanine	%	–
Lupanine	%	–
130H Lupanine	%	–
Sparteine	%	33.60
Lupinine	%	63.29
Oligosaccharides	g/kg DM	8.56
Raffinose	g/kg DM	1.10
Stachyose	g/kg DM	4.94
Verbascose	g/kg DM	2.53
P-phytate	%	0.70

### Growth performance

Ducks were reared to 8 weeks of age, consistently with the generally used technology for rearing broiler ducks. Birds were reared in a semi-intensive system and received feed ad libitum. Total feed intake was recorded each week in each group, and the mean feed intake was calculated. The body weight of birds in all groups was measured each week (WLC 12/F1/R scales, Radwag) with accuracy to the nearest ± 0.2 g. Weekly feed intake (g) and body weight (g) were used to calculate the feed conversion ratio per kg of body weight gain (FCR, %). The mortality rate was calculated at the end of the duck rearing period. Gathered data were used to calculate the European Broiler Index (EBI) from the formula:

$$ {\text{EBI}} = \left[ {{\text{LBW}} \times {\text{SR/AB}} \times {\text{FCR}}} \right] \times {1}00 $$

where: LBW—live body weight at the end of duck rearing (kg), SR—survival rate (%), AB—age of birds (days), FCR—feed intake per kg of body weight (kg).

### Carcass and meat traits

Birds were slaughtered at the age of 8 weeks. Ducks were stunned by electricity, and then their heads were cut off (quick bleeding), according to the requirements. The fasting of birds before slaughter lasted 12 h. Five males and five females were selected from each group, with a body weight close to the mean weight of the same sex individuals in their group. Zoometric traits were measured post-mortem with accuracy to the nearest 1 mm: the length of the abdomen with neck (between the first cervical vertebra and the posterior margin of the ischium), the length of the abdomen (between the shoulder joint and the posterior margin of the ischium), the length of the sternum crest (from the anterior to the posterior margin), the length of the shank (between the ankle joint and the lower posterior surface of the first toe at its base), and the chest circumference (behind the wings, along the anterior margin of the sternum crest and the middle thoracic vertebra). Gathered data were used to calculate body conformation indices (%): compactness index (ratio of chest circumference to abdomen length in cm), massiveness index (body weight in kg to abdomen length in cm), and long-leggedness index (shank length to abdomen length in cm). The carcasses were gutted. Using a pH-meter (CP-401, ELMETRON, with an OSH 2,105 knife electrode) with accuracy to the nearest ± 0.01 pH of breast muscle were done. The electrode was inserted at a 45˚ angle into the right superficial breast muscle. Measurement of carcass pH was after 24 h of cold storage at + 4 °C (pH24). Duck carcasses were dissected at a laboratory using the simplified method described by Ziołecki and Doruchowski^40^. The following elements of carcass were weighed (g): carcass with neck, neck, wings, skin with subcutaneous fat, abdominal fat, breast muscles, leg muscles, carcass remains and offal. Carcass elements were weighed on a WLC 12/F1/R scales (Radwag) with accuracy to the nearest ± 0.2 g, and their proportions in the weight of gutted carcass with neck were calculated in %. The weight of gutted carcass without offal (g) and body weight before slaughter (g) were used for the calculation of the dressing percentage (g) of birds according to the formula:

$$ {\text{Dp}} = \left[ {{\text{weight}}\;{\text{of}}\;{\text{gutted}}\,{\text{carcass}}\,{\text{without}}\;{\text{offal}}\;\left( {\text{g}} \right){\text{/body}}\,{\text{weight}}\,{\text{before}}\,{\text{slaughter}}\;\left( {\text{g}} \right)} \right] \times {1}00 $$

For physicochemical analysis 10 (5 males and 5 females) breast and legs muscles from each group were taken. Each piece was assigned by number. Breast and leg muscles were analysed without skin. The outer right part of breast muscles, leg muscles and skin were analysed for colour using a colorimeter (CR 400, Minolta) in the CIE L* a* b* system^41^. Drip loss from breast and leg muscles was measured. For that purpose, right breast muscles and leg muscles were sampled and weighed on a PS 750/X scales (Radwag) with accuracy to the nearest ± 0.1 g, attached to a special stand and left for 24 h in a cold room at 4 °C, weighed again, and drip loss in % was calculated based on the difference in muscle weight before and after storage^42^. The water-holding capacity of breast and leg muscles was analysed with the Grau and Hamm method^43^. Muscles were disintegrated (K35 mincer, Electrolux), and 280 to 320 mg samples were weighed with accuracy to the nearest ± 0.01 mg (Radwag PS 750/X scales), wrapped in filter paper, placed between two glass plates and kept under 2 kg pressure for 5 min. Samples were weighted again after 5 min. Water-holding capacity (%) was calculated as the ratio of sample weight after pressing to its weight before pressing (mg). Cooking loss from breast and leg muscles (without skin) was analysed. Muscles were disintegrated in a mincer (K35, Electrolux) and 20 g samples were weighed (Radwag PS 750/X). Samples were wrapped in sterile gauze, tied with string, placed in an 80 °C water bath (ADVERTI) for 30 min, and weighed again (PS 750/X). The difference in weight was used to calculate heat-induced leakage from muscles (%). Carcass and meat quality analysis were done according to the methods described by Biesek et al.^9^.

### Fatty acid composition

Samples of subcutaneous and abdominal fat from ducks were preserved, frozen (− 18 °C), lyophilized (Alpha plus apparatus, Donserv) and analysed for fatty acid composition.

Fat was extracted using a technique proposed by Folch et al.^44^, with a mixture of chloroform and methanol (2:1 v/v) on a laboratory shaker. The samples were filtered and left for 24 h for decantation. Fatty acid methyl esters were prepared according to the PN-EN ISO 12,966–2 standard^45^. The fat was dissolved in isooctane and transmethylated with a solution of potassium hydroxide in methanol. Then neutralization of potassium hydroxide with sodium sulphate was carried out. The esters were salted with sodium chloride solution.

Saponified fatty acid esters were separated using a gas chromatograph (type 7,890 B, Agilent Technologies) with an MSD 5977A detector and an autosampler. A capillary column DB-225 MS 60 m × 0.25 mm × 0.25 µm was used for analysis. Analytical parameters were as follows: injection port temperature (split mode 1:100) 230 °C; transfer line temperature 230 °C, ion source temperature 230 °C; quadrupole temperature 150 °C, mode: SIM, ionization type: EI. Oven temperature settings were: 70 °C—increase 0.0 °C/min—hold time 0.0 min; 210 °C—increase 7.0 °C/min—hold time—65.0 min. Carrier gas—helium (flow rate 1.0 ml/min; volume of injected sample 1.0 µl). Fatty acid methyl esters were identified using the Supelco 37 standard FAME Mix component. The individual fatty acids were calculated as the percentage of the total fatty acids identified. Method of fatty acid composition analysis was done similarly, as Kuźniacka et al.^6^ described.

### Statistical analysis

Data were processed using Statistica 12.5 PL software, 2017. Mean values for all analysed parameters and their standard deviations (SD) and coefficients of variation (v) were calculated. A two-way model of ANOVA was used to analyse variability (variable 1—dietary treatment group, variable 2—sex). The significance of differences was verified using the Tukey test. Interactions between experimental variables were determined. The significance of differences was adopted at *P* ≤ 0.05. Production results were calculated for the whole flock of ducks, but the quality of meat traits were calculated for chosen ducks for the slaughter.

### Ethics

The research were done with recommendations of Directive No. 2010/63/EU and resolution 13/2016 of the National Ethics Committee for Animal Experiments of June 17, 2016. The approval of Ethic Committee was not required. The slaughter of birds was carried out in accordance with the applicable rules on the handling of animals at the time of slaughter, including humane treatment. Also the methods used in the meat quality tests were carried out in accordance with the current and commonly used methods described in the Material and methods section.

## References

[1] Ding S, Li G, Chen S, Zhu F, Hao J, Yang F, Hou Z (2019). Comparison of carcass and meat quality traits between lean and fat Pekin ducks. Asian Austral. J. Anim..

[2] Zheng AJ, Chang WH, Hou SS, Zhang S, Cai H, Chen G, Lou R, Liu G (2014). Unraveling molecular mechanistic differences in liver metabolism between lean and fat lines of Pekin ducks (*Anas platyrhynchos domestica*): a proteomic study. J. Proteom..

[3] Zhang Z, Jia Y, Chen Y, Wang L, Lv X, Yang F, He Y, Ning Z, Qu L (2018). Genomic variation in Pekin duck populations developed in three different countries as revealed by whole-genome data. Anim. Genet..

[4] Qamar SH, Zeng Q, Ding X, Bai S, Wang J, Xuan Y, Zhou Q, Su Z, Zhang K (2019). Effect of oil supplementation on growth performance, meat quality and antioxidative ability in meat ducks fed a diet containing aging corn. Int. J. Agric. Biol..

[5] Ao X, Kim IH (2020). Effects of dietary lipid sources on growth performance and carcass traits in Pekin ducks. Poult. Sci..

[6] Kuźniacka J, Banaszak M, Biesek J, Maiorano G, Adamski M (2020). Effect of faba bean-based diets on the meat quality and fatty acids composition in breast muscles of broiler chickens. Sci. Rep..

[7] Rutkowski A, Kaczmarek SA, Hejdysz M, Nowaczewski S, Jamroz D (2015). Concentrates made from legume seeds (*Lupinus angustifolius*, *Lupinus luteus* and *Pisum sativum*) and rapeseed meal as protein sources in laying hen diets. Ann. Anim. Sci..

[8] Hejdysz M, Kaczmarek SA, Rutkowski A (2015). Factors affecting the nutritional value of pea (*Pisum sativum*) for broilers. J. Anim. Feed Sci..

[9] Biesek J, Kuźniacka J, Banaszak M, Adamski M (2020). The quality of carcass and meat from geese fed diets with or without soybean meal. Animals.

[10] Piasecka-Józwiak K, Księżak J, Słowik E, Chabłowska B (2018). The use of lupin flour as nutritional additive to organic wheat sourdough bread. J. Res. Appl. Agric. Eng..

[11] Kaczmarek SA, Hejdysz M, Kubiś M, Rutkowski A (2016). Influence of graded inclusion of white lupin (*Lupinus albus*) meal on performance, nutrient digestibility and intestinal morphology of broiler chickens. Br. Poult. Sci..

[12] Kuźniacka J, Hejdysz M, Banaszak M, Biesek J, Kaczmarek S, Grabowicz M, Rutkowski A, Adamski M (2020). Quality and physicochemical traits of carcasses and meat from geese fed with lupin-rich feed. Animals.

[13] Hang L, Zhang KY, Fraley GS, Ding XM, Bai SP, Wang JP, Peng HW, Zeng QF (2019). High vitamin levels ameliorate negative effect of rapeseed meal in meat ducks by improving antioxidant activity. Poult. Sci..

[14] Recoules E, Lessire M, Labas V, Duclos MJ, Combes-Soia L, Lardic L, Peyronnet C, Quinsac A, Narcy A, Rehault-Godbert S (2019). Digestion dynamics in broilers fed rapeseed meal. Sci. Rep..

[15] Banaszak M, Kuźniacka J, Biesek J, Maiorano G, Adamski M (2020). Meat quality traits and fatty acid composition of breast muscles from ducks fed with yellow lupin. Animal.

[16] Wężyk S, Herbut E, Wawrzycki M (1996). Dobra pasza z „Dobropaszu”. Polskie Drobiarstwo.

[17] Zduńczyk Z, Krawczyk M, Mikulski D, Jankowski J, Przybylska-Gornowicz B, Juskiewicz J (2016). Beneficial effects of increasing dietary levels of yellow lupine (*Lupinus luteus*) seed meal on productivity parameters and gastrointestinal tract physiology in eight-week-old turkeys. Anim. Feed Sci. Technol..

[18] Hejdysz M, Kaczmarek SA, Kubiś M, Jamroz D, Kasprowicz-Potocka M, Zaworska A, Rutkowski A (2018). Effect of increasing levels of raw and extruded narrow-leafed Lupin seeds in broiler diet on performance parameters, nutrient digestibility and AME_N_ value of diet. Anim. Feed Sci..

[19] Kuźniacka, J., Zmudzińska-Pietrzak, A., Roślewska, A., Banaszak, M. & Adamski, M. Effect of native high-protein feeds on the animal products’ quality. Nutritive recommendations due to the use of native high-protein plant feeds for pigs and poultry. Wyd. APRA, Bydgoszcz, pp. 135–151 (2017). (**in Polish**)

[20] Biesiada-Drzazga B, Charuta A, Janocha A, Łęczycka J (2010). Assessment of slaughter value of Pekin STAR 53 HY ducks. Rocz. Nauk. PTZ..

[21] Mierlita D, Popovici D (2013). Effect of partial substitution of soybean meal with lupin seeds on growth and economic efficiency of broilers. Lucrari Stiintifice Seria Zootehnie..

[22] Suchỳ P, Strakova E, Herzig I, Steinahuser L, Vopalensky J, Kroupa L (2010). Effect of replacing soybean meal with lupin seed-based meal in chicken diet on performance, carcass value and meat quality. Acta Vet. Brno..

[23] Kokoszyński D, Bernacki Z (2011). Comparison of slaughter value of Pekin ducks from two conservative flocks. J. Cent. Eur. Agric..

[24] Witak, B., Górski, J. & Górska, A. The effect of yellow lupine meal and extracted rapeseed meal on carcass composition and some characteristics of meat quality of 7-week-old ducks of strain A44. *EPC 2006—12th European Poultry Conference* Verona, Italy, 10–14 September. **13**, 363 (2006).

[25] Kokoszyński D, Wasilewski R, Stęczny K, Bernacki Z, Kaczmarek K, Saleh M, Wasilewski PD, Biegniewska M (2015). Comparison of growth performance and meat traits in Pekin ducks from different genotypes. Eur. Poult. Sci..

[26] Gardzielewska J, Jakubowski M, Karamucki T, Rybarczyk A, Natalczyk-Szymkowska W (2009). Comparison of carcass and meat quality from 17-weeks and 3-years old geese. Rocz. Nauk. PTZ..

[27] Okruszek A, Książkiewicz J, Wołoszyn J, Haraf G, Orkusz A, Szukalski G (2008). Changes in selected physicochemical parameters of breast muscles of geese from Polish conservation flocks depending on duration of the post slaughter period. Arch. Tierz. Dummerstorf..

[28] Pietrzak D, Mierzejewska E, Mroczek J, Michalczuk M, Damaziak K, Makarski M, Adamczak L (2013). Influence of nutrition and sex on the choosen quality traits of meat from White Kołuda geese. Zesz. Probl. Post. Nauk Roln..

[29] Krawczyk M, Mikulski D, Przywitowski M, Jankowski J (2015). The effect of dietary yellow lupin (*L. luteus* cv. Baryt) on growth performance, carcass characteristics, meat quality and selected serum parameters of turkeys. J. Anim. Feed Sci..

[30] Wu P, Wen C, Leng ZX, Zhou YM (2014). Effect of oolong tea (*Camellia sinensis*) powder particle size on growth performance, fat deposition, meat quality and antioxidant activity in meat ducks. Anim. Feed Sci. Technol..

[31] Biesiada-Drzazga B (2006). Analysis of nutrition’ effect on the chemical composition of chosen muscles and fatty acid profile from skin with subcutaneous fat and abdominal fat from broiler geese. Acta Sci. Pol. Zootech..

[32] Biesiada-Drzazga B (2008). Influence of feeding by feed with sunflower meal and yellow lupin on the quality of muscle tissue and fat. Rocz. Inst. Przem. Mięs. Tłuszcz..

[33] Mehaffey JM, Pradhan SP, Meullenet JF, Mekee SR, Owens CM (2006). Meat quality evaluation of minimaly aged broiler breast fillets from five commercial genetic strains. Poult. Sci..

[34] RothMaier DA, Kirchgessner M (1993). Composition and nutritive-value of various white and yellow lupin varieties (*Lupinus-albus* L. and *Lupinus-Luteus* L.) for pigs and poultry. Agribiol Res-Zeitschrift fur Agrarbiologie Agrikulturchemie Okologie..

[35] Mieczkowska A, Smulikowska S (2005). The influence of white lupin seeds in diets supplemented with fats of animal or plant origin on the fatty acid composition of broiler tissues. J. Anim. Feed Sci..

[36] Biesiada-Drzazga, B. The effect of feeding on muscle tissue, skin and fat composition in broiler geese. *Proceedings of XVIIIth International Poultry Symposium PB WPSA “Science for practice—practice for science”.* Rogów, Poland, 266–273 (2006b).

[37] Karpińska M, Batura J (1998). Effect of age, place in organism and sex on the quality of deposited fat in Italian White geese. Zesz. Nauk. Prz. Hod..

[38] Biesiada-Drzazga B, Grużewska A, Janocha A, Markowska M (2010). Analysis of application of concentrated mixtures containing soybean extracted meal and sunflower meal in goose broiler feeding. Arch. Geflugelk..

[39] Kiczorowska B, Samolińska W, Andrejko D (2016). Effect of micronized pea seeds (*Pisum sativum* L.) as a substitute of soybean meal on tissue fatty acid composition and quality of broiler chicken meat. Anim. Sci. J..

[40] Ziołecki, J. & Doruchowski, W. Methods for the evaluation of meat quality and yield. Publisher COBRD Poznań: 1–22 (1989). (in Polish)

[41] CIE. Colorimetry. Publication CIE 15.2. Vienna: Central Bureau of CIE, (1986).

[42] Honikel KO (1987). The water binding of meat. Fleischwirtschaft.

[43] Grau R, Hamm R (1952). Eine einfache Methode zur Bestimmung der Wasserbindung in Fleisch. Fleischwirt..

[44] Folch J, Less M, Stanley GHS (1957). A simple method for the isolation and purification of total lipids from animal tissue. J. Biol. Chem..

[45] PN-EN ISO 12966–2:2011. Vegetable and animal oils and fats. Gas chromatography of fatty acid methyl esters. Preparation of fatty acid methyl esters (2011). (**in Polish**)

